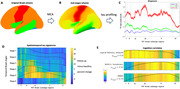# Manifold Component Analysis: a novel technique to spatially profile tau tangles across Alzheimer’s disease stages

**DOI:** 10.1002/alz.092616

**Published:** 2025-01-03

**Authors:** Gleb Bezgin, Tharick Ali Pascoal, Joseph Therriault, Firoza Z Lussier, Stijn Servaes, Min Su Kang, Melissa Savard, Cécile Tissot, Jenna Stevenson, Yi‐Ting Wang, Julie Ottoy, Nesrine Rahmouni, Jaime Fernandez Arias, Arthur C. Macedo, Gassan Massarweh, Paolo Vitali, Jean‐Paul Soucy, Yasser Iturria Medina, Serge Gauthier, Pedro Rosa‐Neto

**Affiliations:** ^1^ McGill University, Montreal, QC Canada; ^2^ University of Pittsburgh, Pittsburgh, PA USA; ^3^ LC Campbell Cognitive Neurology Research Unit, Sunnybrook Research Institute, University of Toronto, Toronto, ON Canada; ^4^ Lawrence Berkeley National Laboratory, Berkeley, CA USA; ^5^ Sunnybrook Research Institute, Toronto, ON Canada; ^6^ McGill University, Montréal, QC Canada; ^7^ Montreal Neurological Institute, McGill University, Montréal, QC Canada

## Abstract

**Background:**

Intracellular accumulation of tau tangles in the brain is one of the most prominent manifestations of Alzheimer’s disease (AD). Progression thereof across the AD stages has specific temporal and spatial patterns, wherein time is informative of space and vice versa. Here we introduce a novel method, Manifold Component Analysis (MCA), to represent tangle accumulation in 2D, reflecting the spatial aspect of tau propagation stages to further relate it to the temporal aspect thereof.

**Method:**

MCA represents a neuroinformatics technique serving to smooth out transitions between neighbouring Braak stage regions (Fig. 1A) and thus creating “sub‐stages” (Fig. 1B) which are subsequently used to sample neocortical (tau tangle) data and represent them in a continuous 2D graph with the spatially earliest sub‐stage appearing in the leftmost, and the latest spatial sub‐stage appearing on the right (Fig. 1C). This method was applied to 18F‐MK‐6240 tau PET tracer data from the TRIAD cohort (https://triad.tnl‐mcgill.com/; N = 753; 123 AD, 170 MCI, 416 cognitively unimpaired).

**Result:**

We obtained MCA profiles of tau‐PET for each subject in the cohort, and evaluated them across multiple visits (Fig. 1D; mean inter‐visit interval 1.9 years), showing that the largest change (about 25%) occurs at stages 4 and 5. Partial correlations between sub‐stages within the MCA profiles for each subject, and their corresponding neuropsychological assessment measurements showed unique spatial signatures for each specific cognitive test, generally favouring earlier stage correlations for memory, and later stage correlations for higher cognition (Fig. 1E); the covariates included age, sex, APOEe4 status and years of education.

**Conclusion:**

The MCA method proves useful as an intuitive lookup tool for tau tangle accumulation across the brain in AD. The obtained profiles help resolve correspondence between “space” and “time” contexts of the pathology spread, as well as the spatial aspect of the timeline of cognitive decline. As MCA is a subject‐specific, easily applicable, fast and extensible technique, numerous new applications and extensions will follow, providing a necessary aid in staging, forecasting, and potentially treating AD.